# Uncovering the origin of the anomalously high capacity of a 3d anode *via in situ* magnetometry†

**DOI:** 10.1039/d2sc06587h

**Published:** 2023-01-04

**Authors:** Xiaoling Teng, Xiangkun Li, Hao Yang, Lu Guan, Yuqi Li, Huiru Yun, Zhaohui Li, Qiang Li, Han Hu, Zhiyu Wang, Mingbo Wu

**Affiliations:** a State Key Laboratory of Heavy Oil Processing, College of Chemistry and Chemical Engineering, China University of Petroleum (East China) Qingdao 266580 P. R. China hhu@upc.edu.cn wumb@upc.edu.cn; b College of Physics, University-Industry Joint Center for Ocean Observation and Broadband Communication, Qingdao University Qingdao 266071 P. R. China liqiang@qdu.edu.cn; c State Key Lab of Fine Chemicals, Liaoning Key Lab for Energy Materials and Chemical Engineering, Dalian University of Technology Dalian 116024 P. R. China zywang@dlut.edu.cn

## Abstract

Transition metals can deliver high lithium storage capacity, but the reason behind this remains elusive. Herein, the origin of this anomalous phenomenon is uncovered by *in situ* magnetometry taking metallic Co as a model system. It is revealed that the lithium storage in metallic Co undergoes a two-stage mechanism involving a spin-polarized electron injection to the 3d orbital of Co and subsequent electron transfer to the surrounding solid electrolyte interphase (SEI) at lower potentials. These effects create space charge zones for fast lithium storage on the electrode interface and boundaries with capacitive behavior. Therefore, the transition metal anode can enhance common intercalation or pseudocapacitive electrodes at high capacity while showing superior stability to existing conversion-type or alloying anodes. These findings pave the way for not only understanding the unusual lithium storage behavior of transition metals but also for engineering high-performance anodes with overall enhancement in capacity and long-term durability.

## Abstract 2

Metallic Co could store lithium *via* a spin-polarized electron injection into its 3d orbital and subsequent electron transfer to the surrounding solid electrolyte interphase, contributing to a totally new lithium storage mechanism.

## Graphical abstract

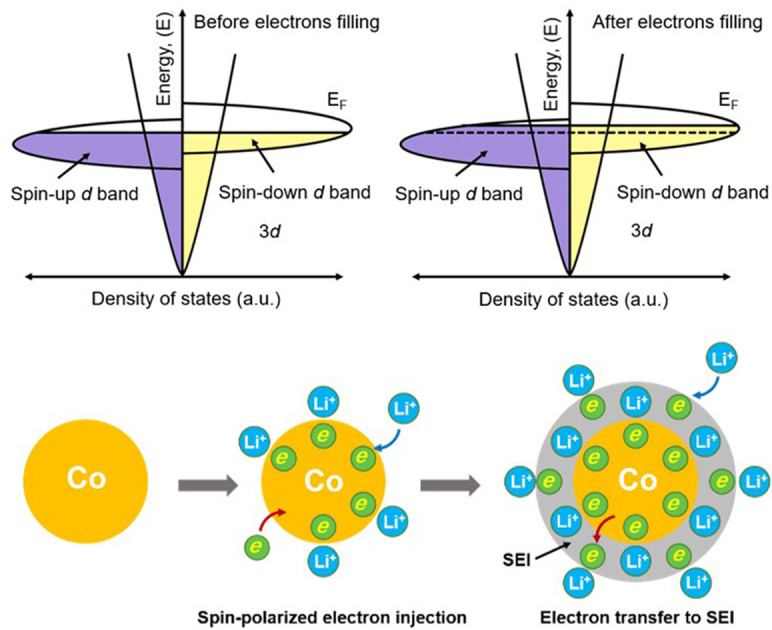

## Introduction

Lithium-ion batteries (LIBs) have become the first choice of power source for portable electronics, but their energy density has fallen behind the demand of high-energy consuming applications.^1^ Approaching an energy target of 500 W h kg^−1^ for long-duration electrical vehicles primarily relies on the development of high-capacity electrodes. For LIBs, the anode materials usually store Li^+^*via* the mechanism of intercalation, pseudocapacitive effect, redox conversion or alloying reactions.^2–4^ The intercalation and pseudocapacitive anodes (*e.g.*, graphite and MXene) work *via* dynamics-controlled reactions to achieve fast lithium storage at the cost of low capacities.^5–9^ Whereas alloying anodes like Si can take up excess Li^+^ to deliver ultrahigh capacities over 4200 mA h g^−1^ but suffer huge volume expansion and fast pulverization.^10–12^ Better electrode stability can be achieved by transition metal compounds (TMCs), which deliver 2–3-fold higher capacities beyond commercial graphite anodes *via* redox conversion to metal nanoparticles dispersed in a Li_2_O matrix.^13–15^ Generally, transition metals with dense lattices are believed inactive for Li^+^ intercalation and alloying with Li. They have been recognized to facilitate lithium storage in TMCs by forming a conductive and buffering matrix, catalyzing redox kinetics and/or the formation of a solid electrolyte interphase (SEI).^16–20^ A variety of anodes have also demonstrated abnormally high capacities,^21–26^ the underlying mechanism for which, however, is still unclear.

For 3d transition metals, the filling state of their spin-up and spin-down d-bands is responsible for the magnetic properties.^27^ Monitoring their magnetic response to electrochemical cycling may give some clues on the evolution of the electronic structure of 3d transition metals. In the Fe_3_O_4_ anode, we have probed the injection of spin-polarized electrons into Fe nanoparticles on the order of Thomas–Fermi screening length by *in situ* magnetometry.^28,29^ This effect creates space charge zones for accommodating extra electrons and Li^+^ on a high density of Fe/Li_2_O interfaces.^30^ As a result, extra capacities beyond the theoretical limit can be delivered by conjunction of charge storage with redox conversion of TMCs. For anodes, however, this mechanism may not solely work for anomalously high capacities due to the absence of metal/Li_2_O boundaries.

Herein, we engaged to uncover the origin of the anomalously high capacity of 3d transition metals for lithium storage *via in situ* magnetometry. A nanostructured Co/C-based anode is taken as a model system to demonstrate the mechanism involved. Such an anode exhibits a two-stage mechanism for lithium storage, namely, the injection of a spin-polarized electron into the unfilled 3d orbitals of metallic Co and the subsequent electron transfer to reduce the surrounding SEI at lower potential. Conjunction of both effects contributes to high capacities of the metallic Co anode. These results highlight the critical role of *in situ* magnetometry in the research of reaction mechanisms of transition-metal based materials. The proposed charge storage mechanism enables fast lithium uptake with capacitive-like behavior without sacrificing high capacity. Meanwhile, it maximizes the electrode stability by avoiding the volume expansion of the electrode encountered by conversion-type and alloying anodes.

## Results and discussion

**Fig. 1:**
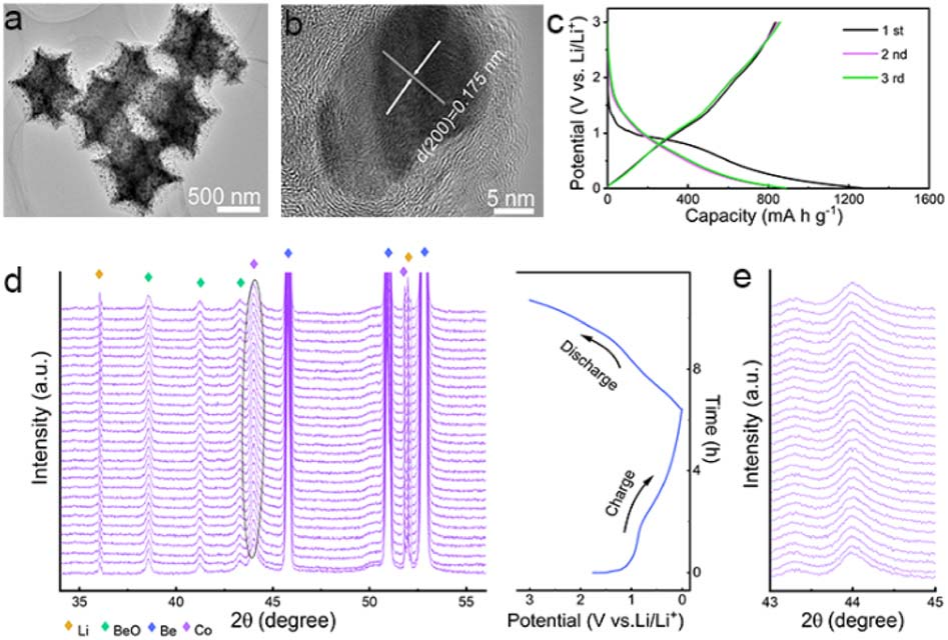
(a) TEM and (b) HRTEM images of the Co/C NPs. (c) GCD curves of the Co/C NPs anode at a current density of 0.1 A g^−1^ between 0.01–3.0 V (*vs.* Li/Li^+^). (d) *In situ* XRD patterns of Co/C NPs anode for the first discharge and charge process. (e) Enlarged view of the diffraction peak from metallic Co in *in situ* XRD patterns.

Zeolitic imidazolate framework-67 (ZIF-67), a typical kind of metal–organic framework (MOF), is used as the precursor to produce the anode materials. Unlike previous research, ZIF-67 nanoparticles, instead of converting into porous carbons, metal compounds, or their hybrids,^31–33^ are directly annealed at a high temperature in inert atmosphere to produce Co/C nanoparticles for lithium storage (Fig. S1, ESI†). As shown, polyhedral ZIF-67 particles are topologically converted to Co/C NPs with a similar shape and a uniform size of *ca.* 800 nm on average (Fig. S2 and S3a, b†). The peaks of ZIF-67 match well with the patterns of the simulated one (Fig. S2c†).^34^ Formation of fcc Co (JCPDS card no. 15-0806) in Co/C NPs is revealed by the peaks at 44.2°, 51.5°, and 75.85° from its (111), (200), and (220) facets in the X-ray powder diffraction (XRD), respectively (Fig. S3c†).^34^ No signals of cobalt oxides are detected by XRD, indicating that only a small amount of metal surface may be oxidized. The dominant metallic state of Co is further validated by a sharp 2p_1/2_/2p_3/2_ doublet at 795.8/780.3 eV in the Co 2p X-ray photoelectron spectroscopy (XPS) spectrum (Fig. S4†).^34,35^ In Co/C NPs, single-crystalline Co nanoparticles with the tiny size of several nanometers are evenly dispersed within an amorphous carbon matrix derived from the organic ligands of ZIF-67 (Fig. 1a and b). The N_2_ adsorption–desorption test reveals a high Brunauer–Emmett–Teller (BET) surface area of 264.2 m^2^ g^−1^ for Co/C NPs with the presence of micro- and meso-pores (Fig. S5†). The porous structure with a large specific surface area would allow more active sites to be directly exposed for better performance.^31,36^ The overall Co content in Co/C NPs is *ca.* 49 wt%, as measured by inductively coupled plasma mass spectrometry (ICP-MS). The magnetic hysteresis loop (MH) curve (Fig. S6a†) shows the characteristics of ferromagnetism at room temperature with the magnetization of 58.6 emu g^−1^, which is consistent with the field cooling and zero-field cooling curves shown in Fig. S6b.† Moreover, the magnetization of Co/C can correspond well to the Co content (Fig. S7†).

In theory, lithium storage by Li^+^ intercalation into the dense lattice of metallic Co or their alloying is infeasible.^37–39^ But the Co/C NPs anode performs well for lithium uptake with slope galvanostatic charge–discharge (GCD) curves except for initial discharge involving SEI formation (Fig. 1c). This phenomenon suggests a pseudocapacitance-dominated character of lithium storage in Co/C NPs.^40–42^ Such an effect usually induces fast lithium storage kinetics *via* charge storage and/or redox reactions on the electrode surface at the cost of low capacity due to a lack of diffusion-controlled bulk reactions. However, the Co/C NPs anode delivers an anomalously high capacity of *ca.* 880 mA h g^−1^ at a current density of 0.1 A g^−1^ between 0.01–3 V (*vs.* Li/Li^+^), which far exceeds the value of the commercial graphite anode. During long cycles, this anode exhibits no capacity decay with a high capacity of over 1000 mA h g^−1^ after 200 cycles at a current density of 0.1 A g^−1^ (Fig. S8†), exhibiting superior performance over other related structures (Table S1†) and showing long-term effectiveness of anomalously high capacity.

*In situ* XRD analysis shows that the fcc Co in Co/C NPs experiences no phase transition throughout the discharge–charge process. This result rules out the possibility of lithium storage by redox conversion or an alloying mechanism (Fig. 1d and e). To identify the role of metallic Co nanoparticles in lithium storage, pure carbon nanocubes without Co were prepared (Fig. S9†). As shown, the carbon nanocubes only deliver a stable lithium storage of around 400 mA h g^−1^ (Fig. S10a and b†), much smaller than the capacity afforded by the Co/C NPs even though these nanoparticles possess a much larger specific surface area (1384.1 m^2^ g^−1^, Fig. S10c†). Apparently, the presence of metallic Co nanoparticles plays a vital role in achieving the anomalously high lithium storage capacities of Co/C NPs. Their contribution to the total capacities is roughly estimated to be *ca.* 55% at a current density of 0.1 A g^−1^ (Fig. S8 and S10a†).

**Fig. 2:**
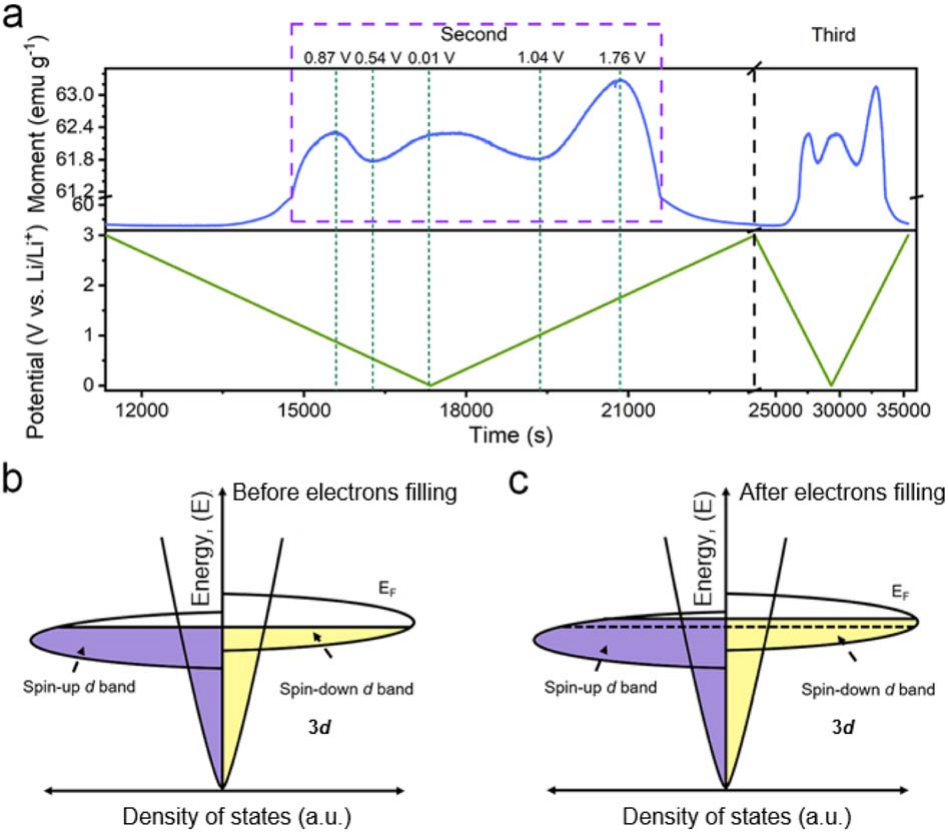
(a) The *in situ* magnetic responses of the Co/C NPs anode at an applied magnetic field of 3 tesla to the second and third cycles at a current density of 0.1 A g^−1^ between 0.01–3.0 V (*vs.* Li/Li^+^). A schematic of the spin-polarized density of states at the surface of ferromagnetic metallic Co grains (b) before and (c) after electron filling into the spin-down d band.

Considering the magnetic response of metallic Co, the magnetometry of Co/C NPs was recorded simultaneously using a specially-designed cell configuration to get an insight into their evolution.^28^ The rationally designed *in situ* cell could deliver a similar performance (Fig. S11†) compared to the widely used coin cells, securing the accuracy of the *in situ* results.^28,43^ The *in situ* magnetometry responses of Co/C NPs are shown in Fig. 2a and S12.† To rule out the influence from other components of the cell, a reference cell without the Co/C NPs anode was also assembled, delivering a constant signal (Fig. S13†), which was subtracted as the background. Meanwhile, the *in situ* magnetometry test was also performed over the carbon nanocube anodes (Fig. S14†), during which the magnetic signal remains unchanged during the charging and discharging processes. In contrast, the magnetic signal changes are particularly pronounced in Co/C NPs. After the initial cycle, the time-dependent potential curves are highly overlapping for the rest of the cycles. A highly reversible magnetic response to the discharge–charge process is simultaneously recorded, which can be divided into four reversible stages. Firstly, the magnetic moment keeps a low level until 1.9 V (*vs.* Li/Li^+^) during discharge, where surface lithium adsorption delivers rather low capacities (Fig. 1c). The magnetization then rapidly rises in a potential range of 1.9 to 0.87 V (*vs.* Li/Li^+^) due to the reduction of surface cobalt oxide to metallic Co (Fig. 2a). According to the quantitative calculation of magnetic measurements, this reduction reaction contributes to a capacity of 25.7 mA h g^−1^, indicating that only a small amount of cobalt is oxidized to cobalt oxide (Fig. S15†). For metallic Co, the ferromagnetism is determined by the different filling states of spin-up and spin-down d bands. They can be correlated with a relationship of *M* = (*N*_↑_ − *N*_↓_)*μ*_B_, where *M* is the net magnetization, *μ*_B_ is the Bohr magneton, and *N*_↑_ and *N*_↓_ are the total number of electrons for each spin. Further discharge of the Co/C NPs anode from 0.87 to 0.54 V (*vs.* Li/Li^+^) induces an unexpected reduction of saturation magnetization. Such a phenomenon indicates the accumulation of more electrons into the spin-down d bands near the Fermi level of Co nanoparticles within a Thomas–Fermi screening length.^30^ It forms space charge zones to accept the electrons while Li^+^ is stored on the particle surface and nearby grain boundaries as a charge balance (Fig. 2c). This spin capacitance effect results in decent extra capacities.^44^ Afterwards, the magnetization of the electrode increases again until the end of discharge at 0.01 V (*vs.* Li/Li^+^). It reveals the transfer of extra electrons from the filled d bands of Co to surrounding SEI if considering the absence of Li_2_O matrix. This electron-injection effect would catalyze the conversion of SEI to radical anions to deliver lithium storage capacities likewise organic electrodes.^45^

During charging, the Co/C NPs anode exhibits an opposite tendency of magnetic response to potential change. The magnetization gradually decreases upon charge to 1.04 V (*vs.* Li/Li^+^). It reflects the electron transfer from radical anions in SEI back to the spin-down d band of Co within a Thomas–Fermi screening length. Subsequent magnetization increases in a potential range from 1.04 to 1.76 V (*vs.* Li/Li^+^) correspond to the extraction of extra electrons from the spin-down d band of Co. Afterwards, the regeneration of the surface oxidized species and Li desorption from the electrode surface gradually decrease the magnetization until the end of charge. Overall, the magnetization of Co/C NPs keeps on a high level above 57 emu g^−1^ before discharge and after charge thanks to a high accumulation of magnetic Co. The asymmetrical variation of magnetization amplitude throughout the discharge–charge process may be a result of asymmetrical electrode reactions and polarization effect.

And then, we further discuss other possible mechanisms that could lead to the changes of the magnetization by transmission electron microscopy (TEM) and MH curves. The valence state of Co remains unchanged in the potential window of 0.01–1.7 V (*vs.* Li/Li^+^) (Fig. S16†), indicating that there is no other mechanism influencing the magnetization.^28,29^ Moreover, the lithium insertion and extraction can influence the exchange interaction and modulate magnetic anisotropy of ferromagnetic nanoparticles,^28,46,47^ leading to magnetization changes in low magnetic fields (Fig. S15†). To eliminate these complications, we took magnetization measurement under high fields above saturation magnetization. Therefore, the proposed spin capacitance and catalytic process are the dominant factors in the magnetic variations instead of other complex chemical reactions, magnetic coupling changes and magnetic anisotropy.

**Fig. 3:**
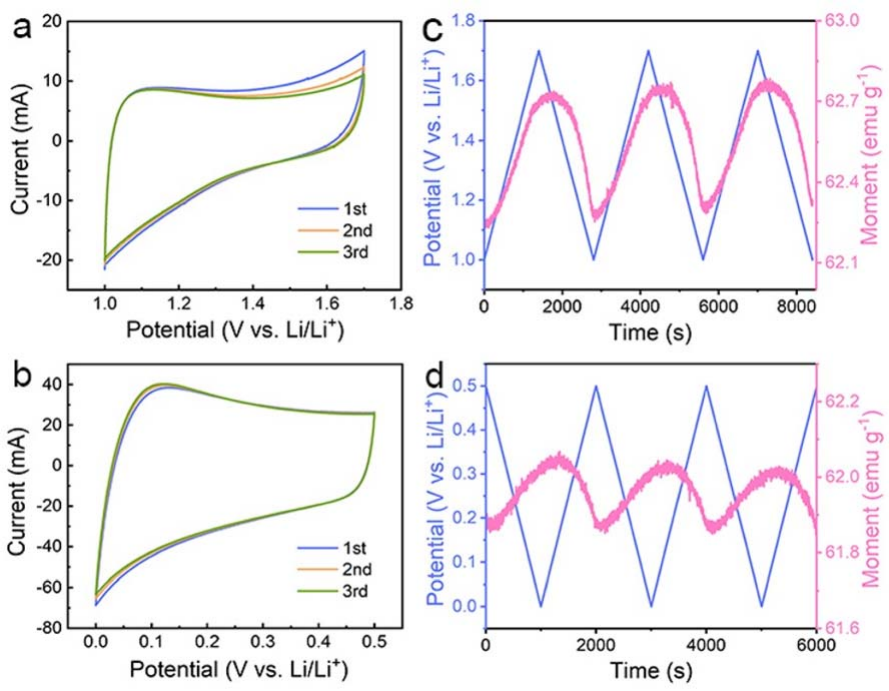
CVs of the Co/C NPs anode at a scan rate of 0.1 mV s^−1^ in a potential range of (a) 1.0–1.7 V (*vs.* Li/Li^+^) and (b) 0.01–0.5 V (*vs.* Li/Li^+^). (c, d) Their corresponding *in situ* magnetic response at an applied magnetic field of 3 tesla.

The above results suggest that the extra capacities of Co nanoparticles for lithium storage may stem from a spin-polarized electron injection into unfilled Co 3d orbitals and electron transfer from Co to the surrounding SEI at lower potentials. To determine the role of the two mechanisms in different potential ranges, the magnetic response of the Co/C NPs anode is further evaluated in a potential range of 1.0–1.7 and 0.01–0.5 V (*vs.* Li/Li^+^), respectively. The former potential range allows one to monitor the extraction of extra electrons from the spin-down d band of Co during charge without the influence of electrode polarization by surface oxides. The Co/C NPs show a typical pseudocapacitive character by rectangular cyclic voltammetries (CVs) without an apparent redox peak between 1.0 and 1.7 V (*vs.* Li/Li^+^) (Fig. 3a). In this region, the magnetization monotonically increases to reach the maximum at 1.7 V (*vs.* Li/Li^+^) with potential rising and *vice versa* (Fig. 3b), reflecting domination of the spin capacitance mechanism.^27,29^ Moreover, we established the thermodynamic modelling for Li^+^ ions storage.^43,48^ In Fig. S17a,† the ln *Q vs. E* curve suggests a slope of expected magnitude (1/*n* ∼0.32) at the range of small *Q* (high potential). The experimental curve linearizes with the expected order of magnitude (Fig. S17b†), which indicates that the interfacial charge storage is dominant in the potential range. In a potential range of 0.01–0.5 V (*vs.* Li/Li^+^), reversible electron exchange between the 3d orbital of Co and the radical anions in the surrounding SEI induces the pseudocapacitive lithium storage accompanied with redox behavior (Fig. 3c). Accordingly, the magnetization variation shows an opposite tendency to potential change due to a negative correlation of net magnetization to the filling state of spin-down d band of Co (Fig. 3d). Unfortunately, owing to the coexistence of the catalytic and spin capacitance effects in low potential and the competitive relationship for electrons, we are unable to perform accurate quantitative calculations for each effect. Despite all this, these results suggest the electron transfer from Co to the surrounding SEI plays a dominant role in this potential range for lithium storage.

**Fig. 4:**
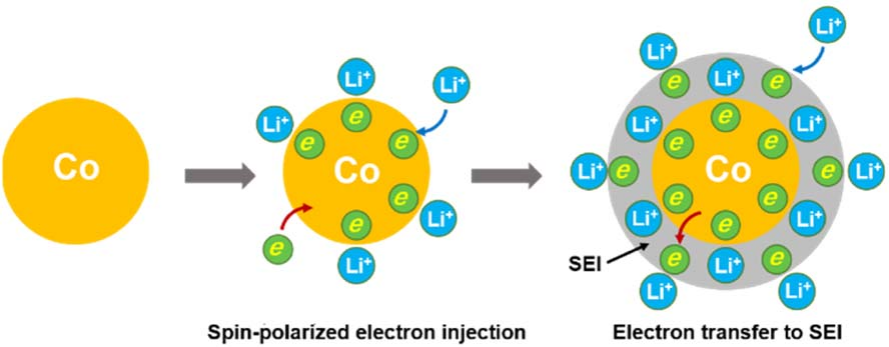
Schematic illustrations of lithium storage in metallic Co *via* a spin-polarized electron injection to the 3d orbital of Co and subsequent electron transfer to the surrounding SEI at lower potential.

Therefore, the lithium storage in metallic Co anode can be primarily described by the following two-stage reversible process (Fig. 4):

1 Co + *x*Li^+^ + *x*e^−^ ↔ *x*Li^+^/Co^*x*−^

2
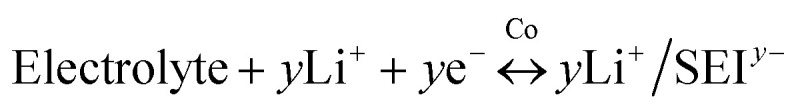


At the first stage, the metallic Co nanoparticles work as a spin capacitor to accumulate electrons into its unfilled d band within a Thomas–Fermi screening length during discharge (eqn (1)). Meanwhile, the Li^+^ ions are taken up onto the particle surface and nearby grain boundaries to balance the space charge, delivering capacities. At a lower potential range, the Co nanoparticles also act as an intermediate to facilitate the electron transfer to the less conductive SEI and reduce it for lithium storage (eqn (2)). Both the surface-controlled processes are critical to induce extra high capacities of metallic Co anode for lithium storage.

**Fig. 5:**
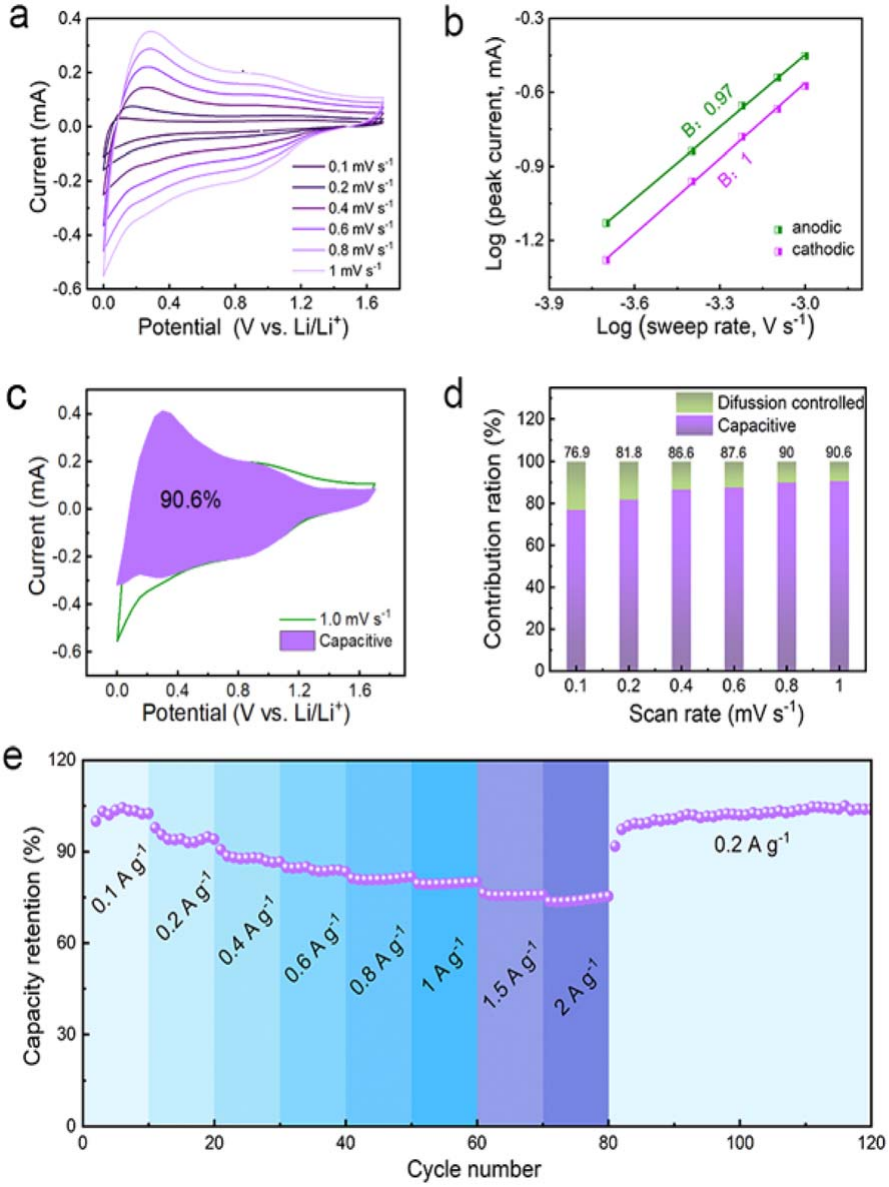
(a) CVs of the Co/C NPs anode at different scan rates of 0.1–1.0 mV s^−1^. (b) The corresponding plots of log(*i*) *vs.* log(*v*) for each redox peak. (c) CVs and the capacitive contribution (purple zone) at a scan rate of 1.0 mV s^−1^. (d) Contribution ratio of capacitive (purple) and diffusion (green) controlled to total capacity at various scan rates. (e) Rate capability performance of Co/C NPs anode.

The unique spin capacitive mechanism allows fast lithium storage in the Co/C NPs anode without sacrificing high capacities, which is distinct from conventional low-capacity pseudocapacitive electrodes. The fast lithium storage kinetics of Co/C NPs are revealed by the constant shape of CVs at different scan rates from 0.1 to 1 mV s^−1^ between 0.01 and 1.7 V (*vs.* Li/Li^+^) (Fig. 5a). The peak current (*i*) and scan rates (*v*) have a relationship of *i* = *av*^*b*^ with *a* and the *b* value of 0.97 and 1.0 for anodic and cathodic processes, respectively (Fig. 5b). This reflects the domination of surface-controlled capacitive behavior for lithium storage.^49,50^ The pseudocapacitance contribution to the total capacity is over 76.9% and increases for a faster scan until a maximum of 90.6% at a scan rate of 1 mV s^−1^ (Fig. 5c). On this basis, electrode kinetics are further analyzed to confirm the capacitance contribution in different potential ranges associated with spin capacitance or SEI-involved reactions. Rectangular CVs appear between 1.0–1.7 V (*vs.* Li/Li^+^) (Fig. S18†) and 0.01–0.5 V (*vs.* Li/Li^+^) (Fig. S19†) at varied scan rates of 0.2 to 1 mV s^−1^. For all cases, the *b* values are 0.99–1.0 throughout the discharge–charge process (Fig. S18b and 19b†), indicating an even dominance of capacitive behavior. Fast electrode kinetics enables a capacity retention as high as 74% when current densities are increased by 20-fold (Fig. 5e), while the specific capacity can even be boosted by switching the current rate back to the initial value. Since the interface between the metallic Co and SEI plays a key role in determining the electrochemical capability, the promoted performance could be attributed to the improved SEI.^18^

Lithium storage based on electron exchange in the Co/C NPs anode brings additional benefits in minimizing the notorious electrode expansion and damage. It enables intrinsic high electrode stability that enhances the conversion-type or alloying anodes suffering from severe volume expansion and pulverization upon lithium uptake. Excellent cycling stability of Co/C NPs is demonstrated by over 100% capacity retention with high coulombic efficiency for 600 cycles at a current rate of 0.5 A g^−1^ (Fig. S20a†), the carbon nanocubes without Co nanoparticles only have 34.7% capacity retention (Fig. S20b†). The post-mortem of the anode after deep cycling shows that the texture of the Co/C NPs is maintained without structural degradation (Fig. S21†). This merit is particularly attractive for manufacturing high-loading thick electrodes and solid-state cells. Such an electrochemical improvement also suggests the long-term effectiveness of spin capacitance and SEI-involved mechanism for efficient lithium storage within metallic Co.

## Conclusions

In summary, the unusual behavior of lithium storage in metallic Co is studied by in situ magnetometry during electrochemical cycling. The origin of the high capacities of nanostructured Co is identified to be as a result of the spin-polarized electron injection effect in conjunction with subsequent electron transfer to the surrounding SEI. Both effects have equally important contributions to the high capacities of 3d transition metals, resulting in capacitive-like lithium storage behavior with fast kinetics but high capacities. The charge storage mechanism without involvement of redox conversion and alloying further avoids electrode destruction, enabling excellent cycling stability for long-term use. This finding may not only provide fresh insight into the understanding of the lithium storage mechanism but also into the development of advanced anodes that can satisfy the criteria of high capacity, fast charge and superior durability.

## Data availability

Essential data are provided in the main text and the ESI.† Additional data are available from the authors upon reasonable request.

## Author contributions

X. Teng, Q. Li and H. Hu conceived the project. X. Teng, X. Li, H. Yun and Y. Li conducted the experiments. H. Yang, L. Guan and Z. Li characterized the samples. X. Teng and X. Li analyzed the data. X. Teng wrote the paper with the help of H. Hu, Q. Li, Z. Wang and M. Wu.

## Conflicts of interest

There are no conflicts to declare.

## Supplementary Material

SC-014-D2SC06587H-s001

## References

[cit1] Armand M., Tarascon J. M. (2008). Nature.

[cit2] Cheng H., Shapter J. G., Li Y., Gao G. (2021). J. Energy Chem..

[cit3] Wang H., Wang C., Tang Y. (2021). EcoMat.

[cit4] Xi F., Zhang Z., Hu Y., Li S., Ma W., Chen X., Wan X., Chong C., Luo B., Wang L. (2021). J. Hazard. Mater..

[cit5] Chae S., Choi S., Kim N., Sung J., Cho J. (2020). Angew. Chem., Int. Ed..

[cit6] Zhao Z., Das S., Xing G., Fayon P., Heasman P., Jay M., Bailey S., Lambert C., Yamada H., Wakihara T., Trewin A., Ben T., Qiu S., Valtchev V. (2018). Angew. Chem., Int. Ed..

[cit7] Zhang J., Han J., Yun Q., Li Q., Long Y., Ling G., Zhang C., Yang Q. (2021). Small Sci..

[cit8] Niu S., Wang Z., Yu M., Yu M., Xiu L., Wang S., Wu X., Qiu J. (2018). ACS Nano.

[cit9] Tang J., Peng X., Lin T., Huang X., Luo B., Wang L. (2021). eScience.

[cit10] Teng X., Zhang F., Li Q., Wang X., Ye W., Li H., Xu J., Cao D., Li S., Hu H. (2020). J. Electrochem. Soc..

[cit11] Sun B., Lou S., Qian Z., Zuo P., Du C., Ma Y., Huo H., Xie J., Wang J., Yin G. (2019). Nano Energy.

[cit12] Xi F., Zhang Z., Wan X., Li Y., Ma H., Chen X., Chen R., Luo B., Wang L. (2020). ACS Appl. Mater. Interfaces.

[cit13] Li Y., Wu F., Qian J., Zhang M., Yuan Y., Bai Y., Wu C. (2021). Small Sci..

[cit14] Han H., Wei Z., Filatov A. S., Carozza J. C., Alkan M., Rogachev A. Y., Shevtsov A., Abakumov A. M., Pak C., Shatruk M., Chen Y., Dikarev E. V. (2019). Chem. Sci..

[cit15] Wang W., Xiong F., Zhu S., Chen J., Xie J., An Q. (2022). eScience.

[cit16] Chen D., Feng C., Han Y., Yu B., Chen W., Zhou Z., Chen N., Goodenough J., He W. (2020). Energy Environ. Sci..

[cit17] Laruelle S., Grugeon S., Poizot P., Dollé M., Dupont L., Tarascon J. M. (2002). J. Electrochem. Soc..

[cit18] Kim H., Choi W., Yoon J., Um J. H., Lee W., Kim J., Cabana J., Yoon W. S. (2020). Chem. Rev..

[cit19] Cbana J., Monconduit L., Larcher D., Palacin M. R. (2010). Adv. Mater..

[cit20] Cao W., Li Q., Yu X., Li H. (2022). eScience.

[cit21] Guo P., Cao L., Wang R., Hu Y., Xu Z., Huang J., Yao C., Guo L., Cheng Y., Li J., Kajiyoshi K. (2020). Adv. Funct. Mater..

[cit22] Zhou K., Hu M., He Y., Yang L., Han C., Lv R., Kang F., Li B. (2018). Carbon.

[cit23] Etacheri V., Hong C., Tang J., Pol V. G. (2018). ACS Appl. Mater. Interfaces.

[cit24] Meng J., Liu X., Niu C., Pang Q., Li J., Liu F., Liu Z., Mai L. (2020). Chem. Soc. Rev..

[cit25] Xia H., Zan L., Qu G., Tu Y., Dong H., Wei Y., Zhu K., Yu Y., Hu Y., Deng D., Zhang J. (2022). Energy Environ. Sci..

[cit26] Liu X., Li Y., Xu X., Zhou L., Mai L. (2021). J. Energy Chem..

[cit27] Xu H., Zhao L., Liu X., Huang Q., Wang Y., Hou C., Hou Y., Wang J., Dang F., Zhang J. (2020). Adv. Funct. Mater..

[cit28] Li Q., Li H., Xia Q., Hu Z., Zhu Y., Yan S., Ge C., Zhang Q., Wang X., Shang X., Fan S., Long Y., Gu L., Miao G. X., Yu G., Moodera J. S. (2021). Nat. Mater..

[cit29] Li H., Hu Z., Xia Q., Zhang H., Li Z., Wang H., Li X., Zuo F., Zhang F., Wang X., Ye W., Li Q., Long Y., Li Q., Yan S., Liu X., Zhang X., Yu G., Miao G. (2021). Adv. Mater..

[cit30] Chen C., Maier J. (2018). Nat. Energy.

[cit31] Peng Y., Bai Y., Liu C., Cao S., Kong Q., Pang H. (2022). Coordin. Chem. Rev..

[cit32] Peng Y., Xu J., Xu J., Ma J., Bai Y., Cao S., Zhang S., Pang H. (2022). Adv. Colloid Interface Sci..

[cit33] Shen M., Ma H. (2022). Coordin. Chem. Rev..

[cit34] Yang Q., Feng C., Liu J., Guo Z. (2018). Appl. Surf. Sci..

[cit35] Zhang C., Lu B., Cao F., Yu Z., Cong H., Yu S. (2018). J. Mater. Chem. A.

[cit36] Mao J., Ye C., Zhang S., Xie F., Zeng R., Davey K., Guo Z., Qiao S. (2022). Energy Environ. Sci..

[cit37] Cheng W., Rechberger F., Ilari G., Ma H., Lin W., Niederberger M. (2015). Chem. Sci..

[cit38] Wu Z., Cheng X., Tian D., Gao T., He W., Yang C. (2019). Chem. Eng. J..

[cit39] Matsuo Y., Taninaka J., Hashiguchi K., Sasaki T., Cheng Q., Okamoto Y., Tamura N. (2018). J. Power Sources.

[cit40] Yan C., Jiang L., Yao Y., Lu Y., Huang J., Zhang Q. (2021). Angew. Chem., Int. Ed..

[cit41] Lee Y. (2021). J. Power Sources.

[cit42] Ren Q., Yu F., Zhang S., Yin B., Wang Z., Ke K. (2019). Electrochim. Acta.

[cit43] Li X., Su J., Li Z., Zhao Z., Zhang F., Zhang L., Ye W., Li Q., Wang K., Wang X., Li H., Hu H., Yan S., Miao G., Li Q. (2022). Sci. Bull..

[cit44] Rondinelli J. M., Stengel M., Spaldin N. A. (2008). Nat. Nanotechnol..

[cit45] Zhang L., Hu P., Zhao X., Tian R., Zou R., Xia D. (2011). J. Mater. Chem..

[cit46] Yang B., Zhang J., Jiang L., Chen W., Tang P., Zhang X., Yan Y., Han X. (2017). Phys. Rev. B.

[cit47] Wang F., Robert R., Chernova N., Pereira N., Omenya F., Badway F., Hua X., Ruotolo M., Zhang R., Wu L., Volkov V., Su D., Key B., Whittingham M., Grey C., Amatucci G., Zhu Y. M., Graetz J. (2011). J. Am. Chem. Soc..

[cit48] Fu L., Chen C., Samuelis D., Maier J. (2014). Phys. Rev. Lett..

[cit49] Wang L., Nie Y., Zhang X., Zhang G., Lu Q., Duan H. (2021). Chem. Eng. J..

[cit50] Dong X., Yang Y., Wang B., Cao Y., Wang N., Li P., Wang Y., Xia Y. (2020). Adv. Sci..

