# Supplementary material for: Uncovering the origin of the anomalously high capacity of a 3d anode via in situ magnetometry
Source: Chem Sci. 2023 Jan 4;14(9):2455–60. doi: 10.1039/d2sc06587h (PMC9977458; doi:10.1039/d2sc06587h)
Supplement: SC-014-D2SC06587H-s001 [file SC-014-D2SC06587H-s001.pdf]

Electronic Supplementary Material (ESI) for Chemical Science.
This journal is © The Royal Society of Chemistry 2023

Supporting Information

Uncovering the Origin of Anomalously High Capacity of 3*d* Transitional Metal Anode via *in-situ* Magnetometry

Xiaoling Teng,[a] Xiangkun Li,[b] Hao Yang,[a] Lu Guan,[a] Yuqi Li,[a] Huiru Yun,[a] Zhaohui Li,[b] Qiang Li,*[b] Han Hu,*[a] Zhiyu Wang,*[c] and Mingbo Wu*[a]

a X. Teng, H. Yang, L. Guan, Y. Li, H. Yun, Prof. H. Hu and Prof. M. Wu

State Key Laboratory of Heavy Oil Processing, College of Chemical Engineering

China University of Petroleum (East China)

Qingdao 266580, China

E-mail: hhu@upc.edu.cn; wumb@upc.edu.cn

b X. Li, Z. Li, and Prof. Q. Li

College of Physics, University-Industry Joint Center for Ocean Observation and Broadband Communication

Qingdao University

Qingdao 266071, China

E-mail: liqiang@qdu.edu.cn

c Prof. Z. Wang

State Key Lab of Fine Chemicals

Liaoning Key Lab for Energy Materials and Chemical Engineering

Dalian University of Technology

Dalian 116024, China

E-mail: zywang@dlut.edu.cn

Table of Contents

Experimental details......1

Figs.

Fig. S1 Schematic illustration of the preparation of Co/C NPs......3

Fig. S2 SEM images and XRD pattern of precursor ZIF-67......3

Fig. S3 SEM images and XRD pattern of Co/C NPs......3

Fig. S4 XPS spectra of Co/C NPs......4

Fig. S5 N_2 adsorption/desorption isotherms of Co/C NPs......4

Fig. S6 MH loop curves and field cooling and zero-field cooling curves of the as-prepared Co/C NPs......4

Fig. S7 TG analysis of the as-prepared Co/C NPs under air atmosphere......5

Fig. S8 Cycling performance of Co/C NPs......6

Fig. S9 XRD pattern, XPS spectra and SEM image of carbon nanocubes......6

Fig. S10 Electrochemical performance and N_2 adsorption/desorption isotherms of carbon nanocubes......6

Fig. S11 GCD curves for the operando magnetometry cell and coin-type cell......7

Fig. S12 The *in-situ* magnetic response of Co/C NPs to electrochemical cycling......7

Fig. S13 Comparison of in-situ magnetic responses of Co/C NPs and without Co/C NPs......7

Fig. S14 The *in-situ* magnetic response of carbon nanocubes to electrochemical cycling......8

Fig. S15 MH curves of Co/C NPs electrode at different potentials......8

Fig. S16 HR-TEM images of the Co/C NPs electrode at different potentials......9

Fig. S17 Thermodynamic calculation of lithium storage......9

Fig. S18 CVs of Co/C NPs anodes at different sweep rates between 1.0 and 1.7 V (vs. Li/Li^+)......10

Fig. S19 CVs of Co/C NPs anodes at different sweep rates between 0.01 and 0.5 V (vs. Li/Li^+)......10

Fig. S20 Cycling performances of Co/C NPs and carbon nanocubes......10

Fig. S21 TEM images of Co/C NPs before and after 350 cycles......11

Table

Table S1 The electrochemical performance comparison for various Co/C nanoparticles and their composites......12

References......13

Experimental details

Synthesis of Co/Carbon Nanoparticles

The $Co(NO_3)_2·6H_2O$ (1 mmol) and 2-methylimidazole (4 mmol) were dispersed in methanol (25 mL) separately to produce two solutions. The solution of 2-methylimidazole was quickly poured into the solution of $Co(NO_3)_2$. After aging for 24 h at room temperature, the purple precipitate was harvested via centrifugation (6,000 rpm, 3 min), washing with methanol for three times, and drying under vacuum at 70 ^oC for 8 h. The obtained zeolitic imidazolate framework-67 (ZIF-67) nanoparticles were annealed at 700 ^oC for 6 h with a ramping rate of 5 ^oC min^{-1} in Ar flow to generate the Co/carbon nanoparticles (Co/C NPs).

Synthesis of Carbon Nanocubes

Typically, $Zn(NO_3)_2·6H_2O$ (1 mmol) and 2-methylimidazole (4 mmol) were dissolved in methanol (50 mL) under stirring at room temperature. The product was collected from the colloidal dispersion by centrifugation (6000 rpm, 3min) and washed with methanol three times. Finally, the obtained products were dried under vacuum at 70 ^oC for 8 h. The obtained ZIFs were annealed at 1200 ^oC for 2 h with a ramping rate of 5 ^oC min^{-1} in Ar flow to generate the carbon nanocubes.

Characterizations

The crystal structure of the Co/C NPs was characterized using X-ray diffraction (XRD) with a high-intensity Cu Kα radiation with a wavelength of 1.5406 Å. The chemical bonding state was analyzed by X-ray photoelectron spectroscopy (XPS) using Thermo Scientific ESCALAB 250XI photoelectron spectrometer. The surface morphology was investigated by scanning electron microscope (SEM; JSM-6700F). Transmission electron microscopy (TEM) and high-resolution transmission electron microscopy (HR-TEM) were carried out by a JEOL 100CX instrument. The thermalgravimetric (TG) analysis were obtained on a thermogravimetric analyzer (PerkinElmer TGA 4000) with a heating rate of 10 ^oC min^{-1} under air atmosphere. Brunauer-Emmett-Teller (BET) specific surface area and pore size were determined from N_2 adsorption/desorption isotherms at 77 K using a Micromeritics ASAP 2020 instrument. The element content was conducted by inductively coupled plasma-optical emission spectroscopy (ICP-OES; Agilent 710).

Electrochemical measurements

The electrochemical performances of the Co/C NPs were measured by two-electrode LIR-2032-type coin cells, which were assembled in an argon-filled glove box with the Co/C NPs as the working electrodes, lithium foils as counter-electrodes and Celgard 2250 films as separators. The working electrodes were prepared by mixing the Co/C NPs, acetylene black (super P), and carboxymethyl cellulose (CMC) in a weight ratio of 7:2:1, and the resulting slurry was coated uniformly on a Cu foil, and dried at 80 ^oC under vacuum for 12 h. The electrolyte solution was 1 M $LiPF_6$ in a 1:1 (w/w) mixture of ethylene carbonate (EC) /dimethyl carbonate (DMC). Charge-discharge tests and galvanostatic charge-discharge (GCD) curves were conducted by a battery test system (NEWARE-4008) at various current rates between 0.01 and 3.0 V vs. Li/Li^+. Cyclic voltammetry (CV) was carried out on a CHI660E electrochemical workstation.

In-situ magnetometry

The Co/C NPs electrode, lithium foil and electrolyte were the same as the typical electrochemical evaluation. The in-situ magnetometry performances of the composite films were measured by a long strip of flexible packaging batteries which were also assembled in an argon-filled glove box. The magnetic properties were probed by a Quantum Design physical property measurement system (PPMS) magnetometer at room temperature. All in-situ magnetization measurements were carried out simultaneously during the electrochemical discharge-charge processes.

Figs.

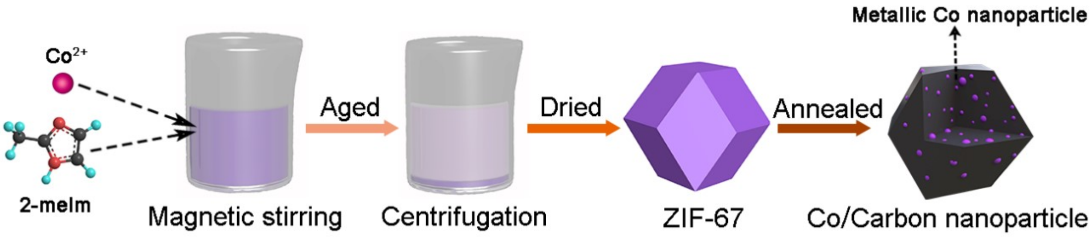

Fig. S1 Schematic illustration of the preparation of Co/C NPs.

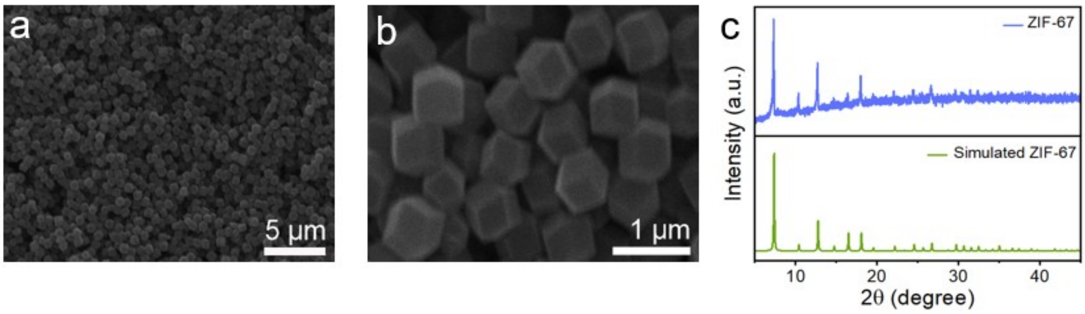

Fig. S2 (a, b) SEM images and (c) XRD pattern of ZIF-67.

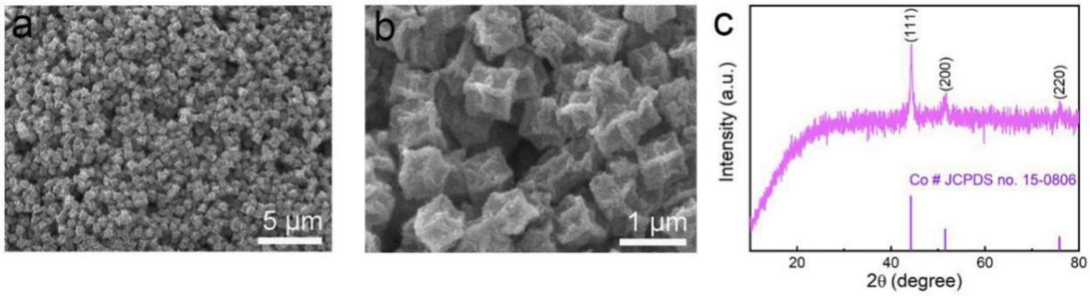

Fig. S3 (a, b) SEM images and (c) XRD pattern of Co/C NPs.

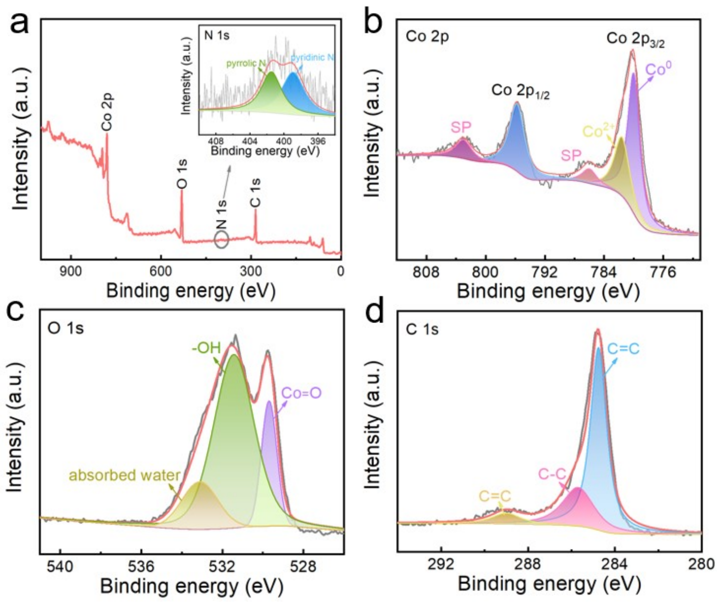

Fig. S4 (a) Full XPS spectra of Co/C NPs and high-resolution spectra of (b) Co 2p, (c) O 1s, (d) C 1s and N 1s (insert in (a)).

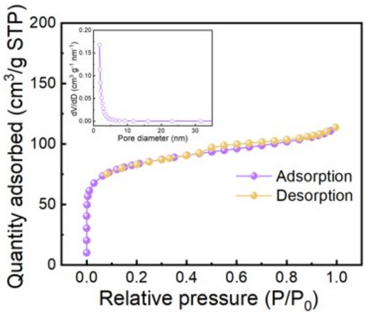

Fig. S5 N_2 adsorption/desorption isotherms of Co/C NPs. Inset is the pore-size distribution.

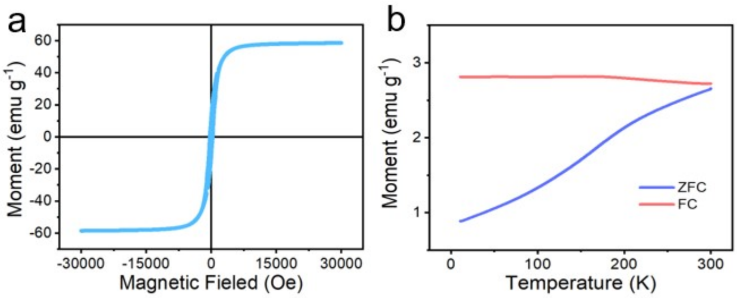

Fig. S6 (a) Magnetic hysteresis (MH) loop curves of the as-prepared Co/C NPs at room temperature. (b) Field cooling and zero-field cooling curves of the as-prepared Co/C NPs.

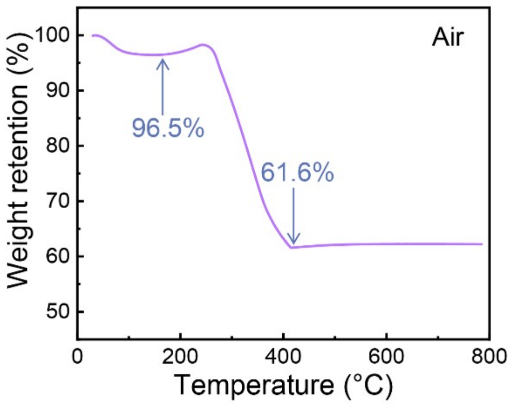

Fig. S7 TG analysis of the as-prepared Co/C NPs under air atmosphere.

Calculations about the magnetization and content of Co.

Firstly, the TG analysis of Co/C NPs were conducted to determine the cobalt content. The first weight-loss step below 200 °C is due to the removal of trace adsorbed water, and the second weight-loss step is attributed to the joint outcome of carbon combustion and oxidation of Co nanoparticles. As carbon components can be completely burned in air, the final product will be only Co_3O_4. The TG result indicated that Co/C NPs remained at 61.6 wt% of its original weight (wt% R = 61.6 wt%) when subjected to heat treatment under air atmosphere. Therefore, the carbon content of Co/C NPs (wt% carbon) can be obtained by:

$$\text{wt\% R} = (1 - \text{wt\% carbon} - \text{wt\% water}) \times \frac{M(\text{Co}_3O_4)}{3 \times M(\text{Co})}$$

where wt% water is the content of trace adsorbed water removed in the first weight-loss step, $M(Co_3O_4)$ represents relative molecular mass of Co_3O_4 (240.7) and M(Co) is relative atomic mass of Co (58.9).

The calculated carbon content is 51.3%, indicating the cobalt content is 48.7%. The cobalt content calculated by TG is quite consistent with the ICP-MS result (49%) in the manuscript.

And then, the metallic Co content was calculated by saturation magnetization (MS). The MS of Co/C NPs is about 58.6 emu g^{-1} (Figure S5a). The low MS value could be mainly originated from the existence of non-magnetic carbon species and the inevitable formation of CoO on the surface of Co.[1-3] Additionally, the MS value of the ferromagnetic metals is influenced by size effect and the crystalline degree.[4-6] Therefore, according to previous reports, the MS of metallic Co is about 136 emu g^{-1}.[7, 8]

We can calculate the metallic Co content as follow:

58.6/136 = 43.1 wt% (the metallic Co in Co/C NPs)

The calculated Co content (43.1 wt%) obtained by MS is close to the experimental result (48.7 wt%) obtained by TG. The slightly difference of the Co content between calculation and experiment can be ascribed to partial oxidation of cobalt. Therefore, the oxidized Co in Co/C NPs can be calculated as:

48.7 wt% - 43.1 wt%=5.6 wt% (the oxidized Co in Co/C NPs)

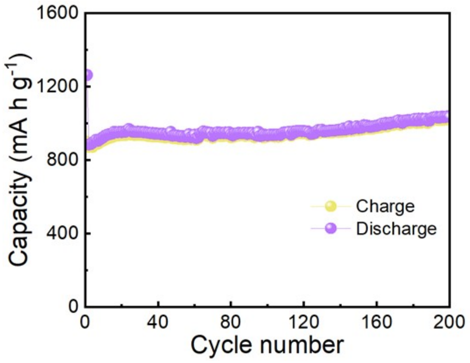

Fig. S8 Cycling performance of Co/C NPs at a current density of 0.1 A g^{-1} between 0.01 to 3 V (vs. Li/Li^{+}).

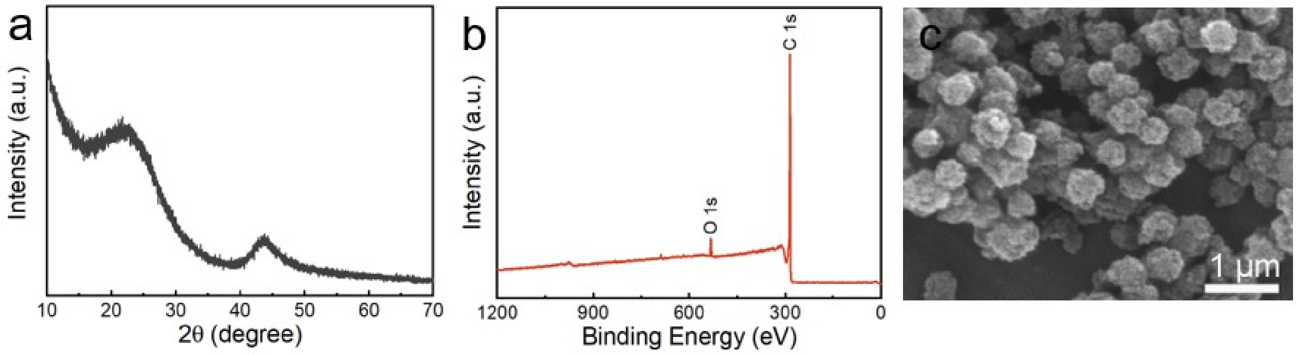

Fig. S9(a) XRD pattern, (b) XPS spectra and (c) SEM image of carbon nanocubes.

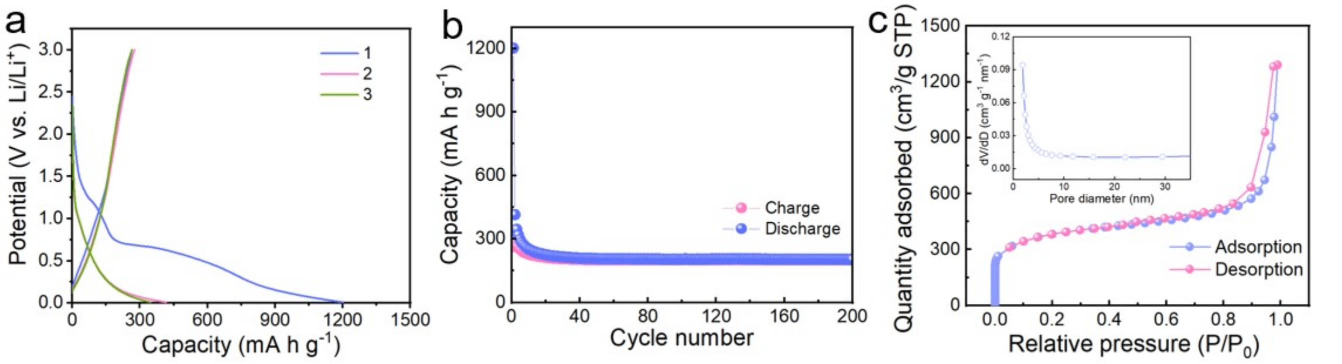

Fig. S10 (a) GCD curves and (b) cycling performance of carbon nanocubes at a current density of 0.1 A g^{-1} between 0.01 to 3 V (vs. Li/Li^{+}). (c) N_2 adsorption/desorption isotherms of carbon nanocubes (inset is the pore-size distribution).

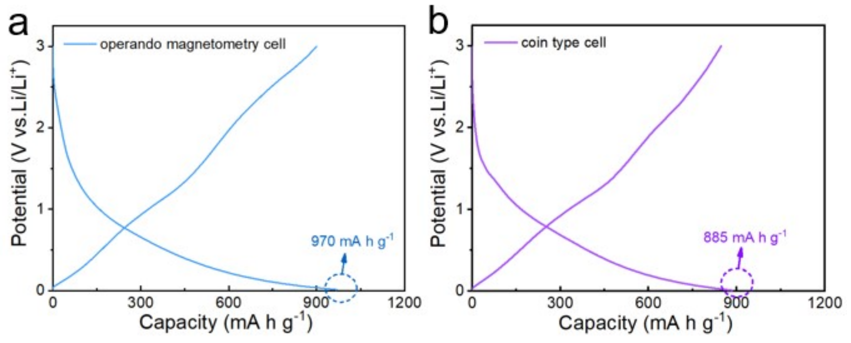

Fig. S11 GCD curves for the (a) operando magnetometry cell and (b) coin-type cell.

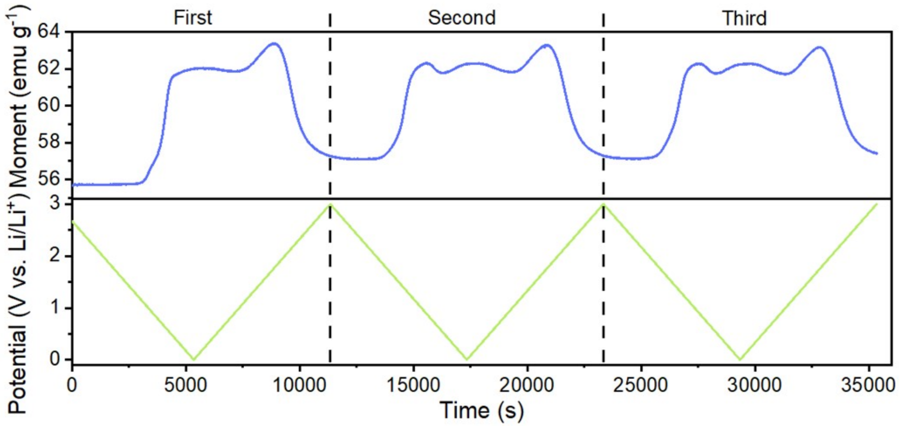

Fig. S12 The *in-situ* magnetic response of Co/C NPs at an applied magnetic field of 3 tesla to electrochemical cycling.

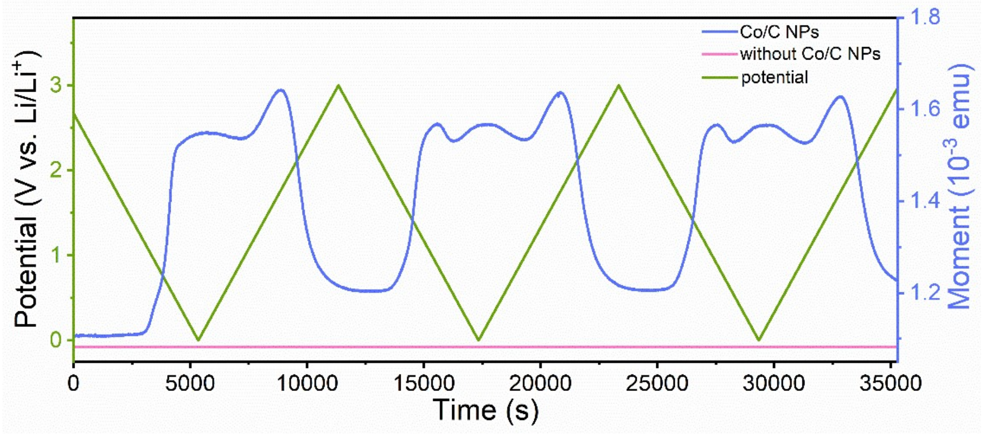

Fig. S13 Comparison of *in-situ* magnetometry magnetic responses of the cell with and without Co/C NPs at an applied magnetic field of 3 tesla.

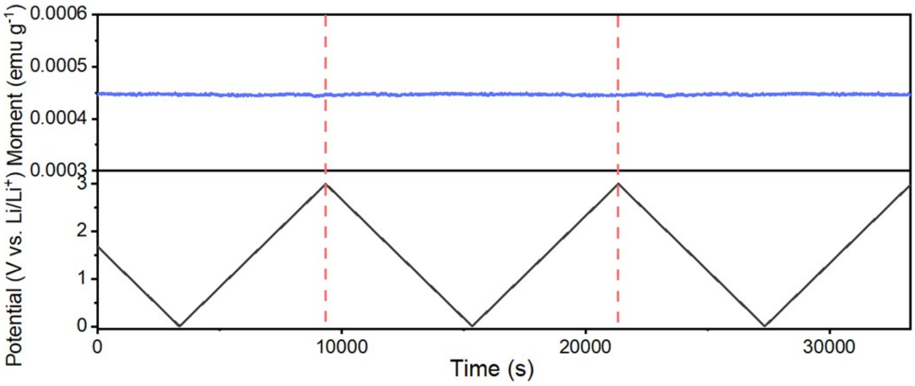

Fig. S14 The *in-situ* magnetic response of carbon nanocubes at an applied magnetic field of 3 tesla to electrochemical cycling.

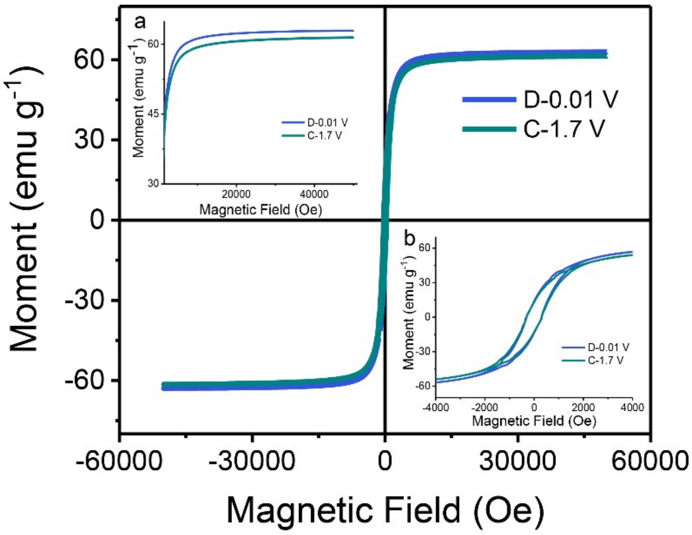

Fig. S15 MH curves of Co/C NPs electrode discharged to 0.01 V (vs. Li/Li^+) and charged to 1.7 V (vs. Li/Li^+). Inset: a and b are magnified views of MH curves at saturated magnetic field and low magnetic field, respectively.

Quantitative calculation of the capacity based on magnetism variation

As shown in Figure S15, the magnetization (m) of Co/C NPs anode discharged to 0.01 V (vs. Li/Li^+) is 62.4 emu g^{-1}. Compared with the pristine electrode (58.6 emu g^{-1}), the m change is Δm_1=3.8 emu g^{-1}, which is fully contributed by reduction reaction of CoO. Considering the magnetization changes caused by the conversion of CoO per unit mass into cobalt is:[8, 9]

$$\Delta m_2 = 136 \times \frac{M(\text{Co})}{M(\text{CoO})} \qquad (\text{emu g}^{-1})$$

where M(Co) is relative atomic mass of Co (58.9) and M(CoO) represents relative molecular mass of CoO (74.9).

For Co/C NPs anode, the mass ratio of CoO (wt% CoO) can be obtained by:

wt% CoO = $\Delta m_1/\Delta m_2$

Therefore, the CoO mass ratio in Co/C anode is 3.6 wt%.

Considering that the theoretical specific capacity of CoO is 715 mA h g^{-1},[10] the capacity contributed by redox processed of CoO in Co/C anodes (Q) can be expressed as:

$$Q = \text{wt\% CoO} \times 715 \qquad (\text{mA h g}^{-1})$$

And then, the capacity contributed by redox processes of CoO can be calculated as 25.7 mA h g^{-1}.

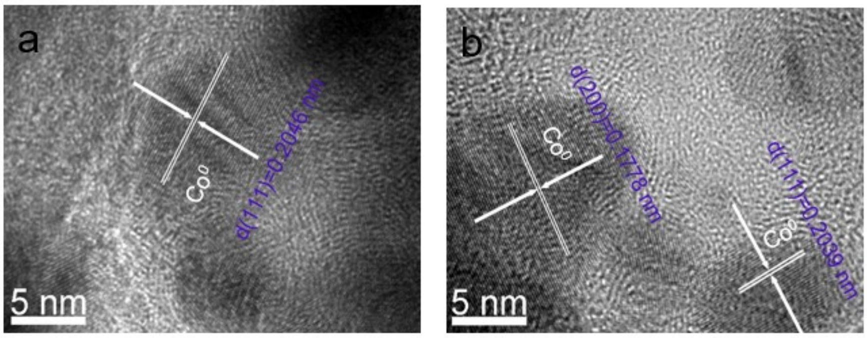

Fig. S16 High-resolution TEM (HRTEM) images of the Co/C NPs electrode discharged to 0.01 V (vs. Li/Li^+) (a) and charged to 1.7 V (vs. Li/Li^+) (b).

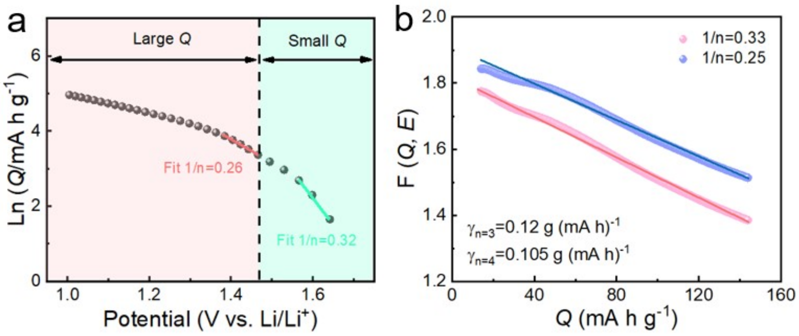

Fig. S17 (a) Dependence of lithium interfacial space charge storage and lithium activity. (b) Dependence of F (*Q, E*) vs. *Q* for the interfacial space charge storage process.

Thermodynamics modeling for Li^+ ions storage [10, 11]

$$\exp\left(-\frac{eE}{k_B T}\right) \propto Q^n \exp(\gamma Q) \quad \text{(n is between 3 and 4)} \qquad (1)$$

e represents the charge quantity per electron, *E* denotes the potential, k_B is Boltzmann constant, *T*=300 K, *Q* (mA h g^{-1}) is the charge per mass of composites and *γ* is constant.

The relationship between potential *E* and *Q* can be obtained by thermodynamic model shown in equation (1). For relatively small *Q* (high potentials), the factor Q^n in above equation will dominate, and for large *Q* (low potentials), the factor exp(*γQ*) will dominate. Note that the behavior at high potentials (small *Q*) then emphasizes the diffuse double layer part, while the behavior at low potentials (large *Q*) corresponds to an electrostatic capacitance (potential ∝ *Q*).

Therefore, the equation (1) can be divided into two parts.

For small *Q* (high potentials): $$-\frac{eE}{k_B T} = n\ln Q \qquad (2)$$

For large *Q* (low potentials): $$-\frac{eF(Q,E)}{k_B T} = \gamma Q \qquad (3)$$

In equation (3), $F(Q,E) = [E + n(\frac{k_B T}{e})\ln Q]$.

For the high potential range in Fig. S17a, the fitting results were obtained by equation (2). And then, the low potential range is fitted by equation (3) with the boundary condition (n = 3 or 4) (Fig. S17b). If the fitting *γ* result is close to the theoretical value (is roughly on the order of 1), it means that space charge storage mechanism is dominant within this potential range of 1.0-1.7 V (vs. Li/Li^+).

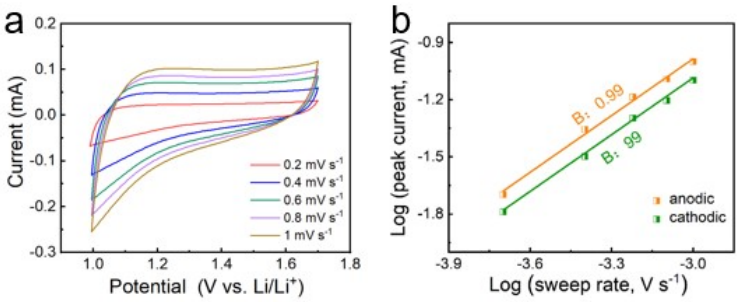

Fig. S18 (a) CVs of Co/C NPs anodes at different sweep rates from 0.2 to 1.0 mV s^{-1} between 1.0 and 1.7 V (vs. Li/Li^+). (b) Determination of the b-value using the relationship between peak current and sweep rate.

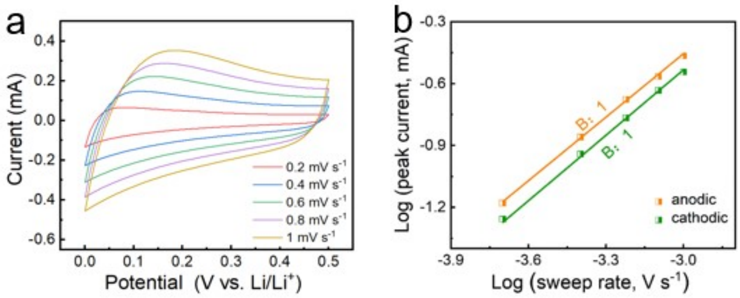

Fig. S19 (a) CVs of Co/C NPs anodes at different sweep rates from 0.2 to 1.0 mV s^{-1} between 0.01 and 0.5 V (vs. Li/Li^+). (b) Determination of the b-value using the relationship between peak current and sweep rate.

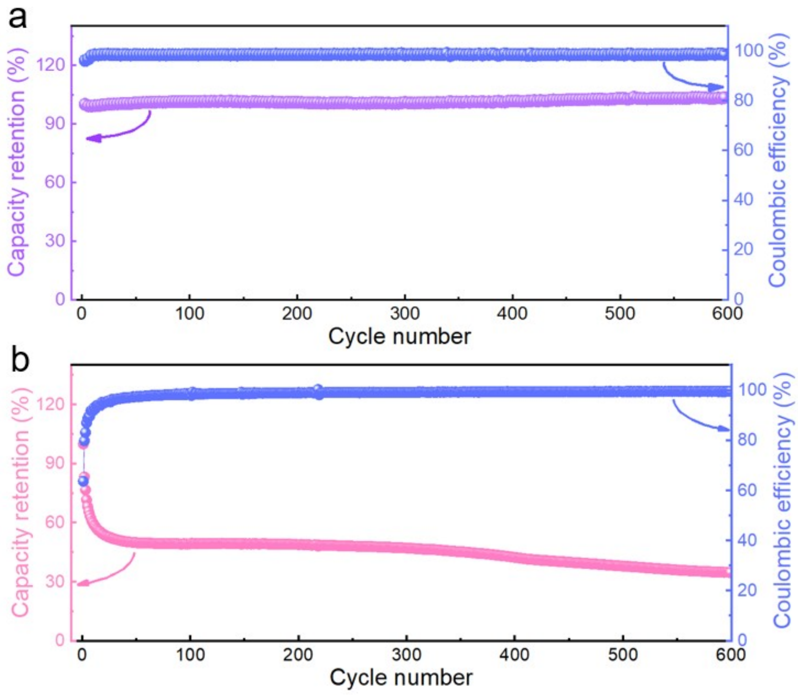

Fig. S20 Cycling performance of (a) Co/C NPs and (b) carbon nanocubes anode at a current density of 0.5 A g^{-1} between 0.01 to 1.7 V (vs. Li/Li^+). The batteries discharged to 0.01 V (vs. Li/Li^+) and charged to 1.7 V (vs. Li/Li^+) firstly, followed by a long cycle test in the potential range of 0.01-1.7 V (vs. Li/Li^+).

For easy comparison, all the cycling performances were conducted in the potential range of 0.01-1.7 V (vs. Li/Li^+) as the Co/C NPs were evaluated in this range. Because of the lithium storage mechanism involving Co nanoparticles mainly occurs in this potential range. Since the open circuit potential of these two batteries is larger than 1.7 V (vs. Li/Li^+). The cycling performance for both samples was recorded since the second cycle.

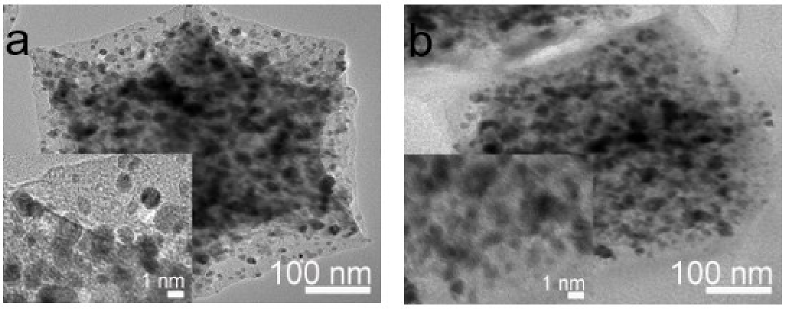

Fig. S21 TEM images of Co/C NPs (a) before and (b) after 350 cycles. The inserts are the HRTEM images of Co NPs.

Table

Table S1 The electrochemical performance comparison for various Co/C nanoparticles and their composites.

Materials	Long cycle Capacity (mAh g^{-1})	Rate performance (C or mAh g^{-1})
N-Doped Co/C Nanocubes[12]	688.6@100 mA g^{-1}@100 cycles	467.6@1000 mA g^{-1}
S-doped Co/N/C mesoporous nanorods[13]	714.8@200 mA g^{-1}@300 cycles	439@2000 mA g^{-1}
Co_3O_4@Co@graphitic carbon[14]	686@200 mA g^{-1}@60 cycles	493@1000 mA g^{-1}
CoSe/Co@N-doped graphitic carbon[15]	630@200 mA g^{-1}@100 cycles	265@2000 mA g^{-1}
CoC_2O_4@CoO/Co composite arrays[16]	802.5@200 mA g^{-1}@200 cycles	553.3@1600 mA g^{-1}
Sb_2O_5/Co-containing carbon polyhedra[17]	1009@100 mA g^{-1}@100 cycles	647.2@1000 mA g^{-1}
Co–CoO_x nanowire arrays[18]	999@10000 mA g^{-1}@1000 cycles	827@2000 mA g^{-1}
Co–Zn/N–C polyhedral nanocages[19]	702@200 mA g^{-1}@400 cycles	444@2000 mA g^{-1}
SnO_2/Co@C nanocubes[20]	800@200 mA g^{-1}@100 cycles	400@5000 mA g^{-1}
Co_3O_4/Co/carbon nanocages composites[21]	801@100 mA g^{-1}@60 cycles	485@5000 mA g^{-1}
Co nanoparticles@porous carbon[22]	950@100 mA g^{-1}@140 cycles	441@2000 mA g^{-1}
Co/CoS_2 nanoparticles/carbon nanotubes[23]	~960@0.1 C@140 cycles	384@2 C
Co_3O_4@carbon nanoboxes[24]	1120@100 mA g^{-1}@100 cycles	420@5000 mA g^{-1}
porous Co_3O_4 hollow dodecahedra[25]	780@100 mA g^{-1}@100 cycles	850@900 mA g^{-1}
Co/C NPs (our work)	1134.7@100 mA g^{-1}@360 cycles	671@2000 mA g^{-1}

References

1 H. F. Cheng, N. L. Yang, X. Z. Liu, Y. L. Guo, B. Liu, J. H. Yang, Y. Chen, B. Chen, Z. X. Fan, Q. P. Lu, S. J. Yuan, J. L. Wang, L. Gu, H. Zhang, *Adv. Mater.*, 2020, **33**, 2007140.

2 N. L. Torad, M. Hu, S. Ishihara, H. Sukegawa, A. A. Belik, M. Imura, K. Ariga, Y. Sakka, Y. Yamauchi, *Small*, 2014, **10**, 2096-2107.

3 Y. Fei, M. Liang, L. W. Yan, Y. Chen, H. W. Zou, *Chem. Eng. J.*, 2020, **392**, 124815.

4 R. Qiang, Y. C. Du, H. T. Zhao, Y. Wang, C. H. Tian, Z. G. Li, X. J. Han, P. Xu, *J. Mater. Chem. A*, 2015, **3**, 13426-13434.

5 Y. Y. Lu, Y. T. Wang, H. L. Li, Y. Lin, Z. Y. Jiang, Z. X. Xie, Q. Kuang, L. S. Zheng, *ACS Appl. Mater. Interfaces*, 2015, *7*, 13604-13611.

6 S. N. Khanna, S. Linderoth, *Phys. Rev. Lett.*, 1991, **67**, 742-745.

7 D. M. Clifford, C. E. Castano, J. V. Rojas, *Radiat. Phys. Chem.*, 2018, **144**, 111-115.

8 W. Liu, Q. W. Shao, G. B. Ji, X. H. Liang, Y. Cheng, B. Quan, Y. W. Du, *Chem. Eng. J.,* 2017, **313**, 734-744.

9 H. Kim, W. Choi, J. Yoon, J. H. Um, W. Lee, J. Kim, J. Cabana, W. S. Yoon, *Chem. Rev.,* 2020, **120**, 6934-6976.

10 L. Fu, C. Chen, D. Samuelis, J. Maier, *Phys. Rev.* Lett.*,* 2014, **112**, 208301.

11 X. Li, J. Su, Z. Li, Z. Zhao, F. Zhang, L. Zhang, W. Ye, Q. Li, K. Wang, X. Wang, H. Li, H. Hu, S. Yan, G. Miao, Q. Li, *Sci. Bull.*, 2022, **67**, 1145-1153.

12 L. Wang, Z. Wang, L. Xie, L. Zhu, X. Cao, *ACS Appl. Mater. Interfaces,* 2019, **11**, 16619-16628.

13 W. She, J. Wang, X. Zhang, M. Sun, C. Xie, J. Xiao, S. Wang, *J. Power Sources*, 2018, **401**, 55-64.

14 Z. Yan, Q. Hu, G. Yan, H. Li, K. Shih, Z. Yang, X. Li, Z. Wang, J. Wang, *Chem. Eng. J.*, 2017, **321**, 495-501.

15 Y. Zhou, R. Tian, H. Duan, K. Wang, Y. Guo, H. Li, H. Liu, *J. Power Sources*, 2018, **399**, 223-230.

16 Z. S. He, L. A. Huang, J. F. Guo, S. E. Pei, H. B. Shao, J. M. Wang, *Energy Storage Mater.*, 2019, **24**, 362-372.

17 J. F. Li, L. Han, X. J. Zhang, G. Zhu, T. Q. Chen, T. Lu, L. K. Pan, *Chem. Eng. J.*, 2019, **370**, 880-809.

18 L. Zhan, S. Q, Wang, L. X. Ding, Z. Li, H. H. Wang, *J. Mater. Chem. A*, 2015, **3**, 19711-19717.

19 M. Huang, K. Mi, J. H. Zhang, H. L. Liu, T. T, Yu, A. H. Yuan, Q. H. Kong, S. L. Xiong, *J. Mater. Chem. A*, 2017, **5**, 266-274.

20 Q, He, J. S, Liu, Z. H. Li, Q. Li, L. Xu, B. X. Zhang, J. S. Meng, Y. Z. Wu, L. Q. Mai, *Small*, 2017, **13**, 1701504.

21 K.Q. Zhou, L. F. Lai, Y. C. Zhen, Z. S. Hong, J. H. Guo, Z. G. Huang, *Chem. Eng. J.*, 2017, **316**, 137-145.

22 H. R. Xu, L. L. Zhao, X. M. Liu, Q. H. Huang, Y. Q. Wang, C. X. Hou, Y. Y. Hou, J. Wang, F. Dang, J. T. Zhang, *Adv. Funct. Mater.*, 2020, **30**, 2006188.

23 X. K. Song, S. Chen, L. L. Guo, Y. Sun, X. P. Li, X. Cao, Z. X. Wang, J. H. Sun, C. Lin, Y. Wang, *Adv. Energy Mater.*, 2018, **8**, 1801101.

24 Y. Huang, Y. J. Fang, X. F. Lu, D. Y. Luan, X. W. Lou, *Angew. Chem. Int. Ed.*, 2020, **59**, 19914-19918.

25 R. B. Wu , X. K. Qian , X. H. Rui , H. Liu , B. L. Yadian , K. Zhou , J. Wei , Q. Y. Yan, X. Q. Feng , Y. Long , L. Y. Wang , Y. Z. Huang, *small*, 2014, **10**, 1932-1938.
